# Correspondence

**Published:** 1889-04

**Authors:** 


### CORRESPONDENCE.

PEYTONVILLE, ARK., April 4, 1889.

*Editor Daniel's Texas Medical Journal:*

Enclosed find $—— subscription to the JOURNAL, and a hastily prepared article for its pages.

You are doing a grand work for the profession in Texas in many ways. Especially I approve of your advocacy of procuring a charter for the State Association. We here in Arkansas feel the need of just such a live and progresssve journal as yours.

We had a good journal, but for want of proper and sufficient support, it went the way of all such; died of starvation, in fact. It is truly said : "We never miss the water until the well runs dry." We only realized the value of the journal, when it was too late to recall it. Under its brief influence the State Society was organized, and the medical department of our University was established; both of which institutions are still living, but feel sadly the want of support of a live journal. I would like *so much* to be with you at San Antonio, for well I know there are many good things in store for those whose good fortune it will be to be present.

While I am doing very well here professionally and otherwise, still I long to be back in dear old Texas—the home of my childhood and early manhood, where I can mingle as of yore with my brethren, and meet with the societies, whose sessions were always so refreshing and so beneficial. I long for them as the hart for the brook; and sigh to think it may be I shall never again drink at their Pierian springs. Keep a look-out for a good place, and —it may be— —.

Well ! With best wishes,

Yours fraternally,

